# The photosensitizer verteporfin has light-independent anti-leukemic activity for Ph-positive acute lymphoblastic leukemia and synergistically works with dasatinib

**DOI:** 10.18632/oncotarget.11025

**Published:** 2016-08-02

**Authors:** Takanobu Morishita, Fumihiko Hayakawa, Keiki Sugimoto, Mizuho Iwase, Hideyuki Yamamoto, Daiki Hirano, Yuki Kojima, Naoto Imoto, Tomoki Naoe, Hitoshi Kiyoi

**Affiliations:** ^1^ Department of Hematology and Oncology, Nagoya University Graduate School of Medicine, Nagoya, Japan; ^2^ Fujii Memorial Research Institute, Otsuka Pharmaceutical Co., Ltd., Otsu, Japan; ^3^ Department of Analytical Neurobiology, Faculty of Pharmacy, Meijo University, Nagoya, Japan; ^4^ National Hospital Organization Nagoya Medical Center, Nagoya, Japan

**Keywords:** drug screening, patient-derived xenograft, Ph^+^ ALL, verteporfin, drug repositioning

## Abstract

Cell lines have been used for drug discovery as useful models of cancers; however, they do not recapitulate cancers faithfully, particularly from the viewpoints of microenvironmental independence. Patient-derived xenografts (PDX) are established by the transfer of primary tumor cells directly from patients into immunodeficient mice and can provide primary-like tumor cells of the amount needed at the desired time. We developed a high-throughput drug screening system using PDX cells and performed drug screening using the PDX cells of Philadelphia chromosome-positive acute lymphoblastic leukemia (Ph^+^ ALL). We established four Ph^+^ ALL PDX mice and performed high-throughput screening of 3440 compounds using leukemia cells from the PDX mice (PDX-cell screening). The profiles of drugs selected by PDX-cell screening were markedly different from those by screening using the Ph^+^ ALL cell line. We found that verteporfin, an FDA-approved drug, exhibited strong PDX cell-specific cytotoxicity. In the validation assay, its GI_50_ was 228 nM, 395 nM, and 538 nM in three PDX cells and 3.93 μM, 2.11 μM, and 5.61 μM in three cell lines. Although verteporfin is a photosensitizer activated by photoirradiation, its cytotoxic effects were mediated by the light-independent production of reactive oxygen species; therefore, its anti-leukemic effects were also exerted *in vivo* without photoirradiation. Furthermore, it exhibited synergistic effects with dasatinib, an ABL kinase inhibitor. These results indicated the potential of verteporfin as a new anti-leukemic reagent.

## INTRODUCTION

ABL kinase inhibitors are highly effective for BCR-ABL-positive leukemias such as chronic myeloid leukemia (CML) and Philadelphia chromosome-positive acute lymphoblastic leukemia (Ph^+^ ALL). ABL kinase inhibitors keep CML in the chronic phase for a long period of time and markedly increase the complete remission rate of Ph^+^ ALL; however, the relapse of Ph^+^ ALL is almost inevitable. [1] The only way to currently achieve a cure is to undergo allogeneic-stem cell transplantation (allo-SCT) during remission [2, 3]; however, the therapy-related death rate of allo-SCT is high, and Ph^+^ ALL is common among the elderly, who cannot receive allo-SCT. Therefore, there are still unmet medical needs for Ph^+^ ALL.

Target-based screening and phenotype-based screening represent the two major ways to develop drugs. In target-based screening, the target molecule of a certain disease, such as a particular kinase, is initially selected based on its knowledge of molecular pathology. Compounds are screened by evaluating their inhibitory effects on the enzymatic activity of the target molecule *in vitro*. Their effects on cell lines and mouse models are then investigated. This is a powerful method that is currently being used to develop many tyrosine kinase inhibitors including ABL kinase inhibitors; however, difficulties have been associated with identifying the true molecular targets of diseases in many cases, and inhibition of the target molecule does not always lead to anti-tumor effects. Even when the target molecule is successfully inhibited, anti-tumor effects may not be obtained due to the activation of an escape signal or evolution of resistant clones with drug-resistant mutations. [4–6] Clonal evolution by the T315I mutation in BCR-ABL represents a severe obstacle for Ph^+^ ALL therapy. [7]

Phenotype-based screening using cell lines as cancer models have widely been used in the development of anti-tumor drugs. In this method, library compounds are added to the culture media of cell lines and selected based on their growth inhibitory effects on the cell lines. Most anti-cancer agents used in standard therapy for leukemia were developed by the 1970s. The development of innovative agents to replace previous standard therapies for leukemia using phenotype-based screening has not advanced for more than 30 years, suggesting the limitations of cell line-based screening and existing libraries for drug development. [8] Completely new libraries and novel cancer models to replace cell lines are now required for the serial development of innovative anti-cancer agents.

In order to overcome these limitations, we previously developed a new high-throughput drug-screening system using lymphoma cells obtained from patient-derived xenografts (PDX). PDX are established by the transfer of primary cancer cells directly from patients into immunodeficient mice. PDX faithfully maintain the characteristics of the parental tumors such as gene expression profiles, the mutational status including genome copy number variants, and metastatic potential even after several passages [9, 10]. PDX also maintain hierarchy of the cancer cell differentiation status [11, 12] and drug responsiveness. [13] Furthermore, PDX may provide the required amount of primary-like cancer cells at the desired time. In the present study, we performed high-throughput screening of 3440 compounds using leukemia cells from Ph^+^ ALL PDX (PDX-cell screening), and found that the photosensitizer, verteporfin, exhibited strong anti-leukemic effects both *ex vivo* and *in vivo*. These effects were mediated by oxidative stress and had synergism with dasatinib, an ABL kinase inhibitor clinically used for Ph^+^ ALL. These results indicated the potential of verteporfin as a new anti-leukemic reagent and PDX-cell screening as a novel strategy for the development of anti-cancer drugs.

## RESULTS

### Establishment of *ex vivo* culturing of PDX cells

Primary Ph^+^ ALL cells obtained from the bone marrow of four patients were intravenously transplanted into NOD/SCID/IL-2Rγ^null^ (NOG) mice. Patients' backgrounds and disease characteristics are summarized in Supplemental Table 1. All leukemia cells were successfully engrafted into mice. A total of 1.3 × 10^8^ to 5.8 × 10^8^ cells were obtained from one PDX mouse and the ratios of leukemia cells were 86.0 to 95.7 % (Supplemental Table 2). PhLO cells were the most efficiently obtained cells.

**Figure 1:**
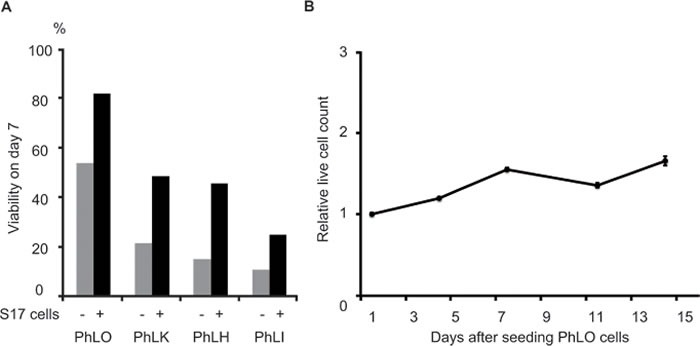
Establishment of an *ex vivo* culture of PDX cells **A.** Survival improvements in PDX cells by a co-culture with stromal cells. PDX cells were cultured with or without S17 cells, as indicated. Viabilities were measured by DAPI staining and a flow cytometric analysis on day 7. **B.** The slow growth rate of PhLO cells *ex vivo*. PhLO cells were cultured with S17 cells as in **A.**. Live cell counts relative to that on day 1 were plotted on a line graph. Assays were performed in triplicate. Mean values were plotted with standard deviations.

PDX cells did not survive well without stromal cells *ex vivo*, and S17 cells, murine bone marrow stromal cells, were found to support the survival of Ph^+^ ALL PDX cells in our previous study. [14] Therefore, we examined the survival of the PDX cells obtained in a co-culture with S17 cells. PhLO cells showed the highest survival rate (Figure 1A) and a slow growth rate with a doubling time of more than 14 days (Figure 1B).

### Verteporfin was specifically selected by PDX-cell screening

**Figure 2:**
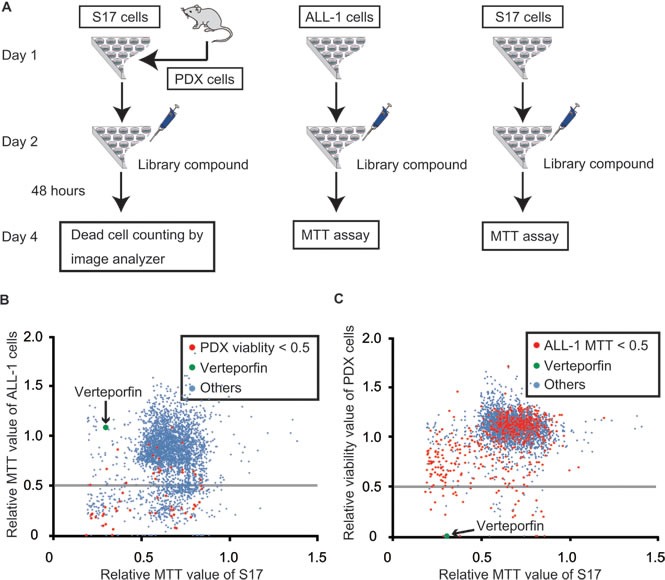
Comparison between PDX-cell screening and Cell-line screening **A.** Flow chart of screenings. PhLO were sacrificed to obtain PDX cells on day 1. PDX cells (1.0 × 10^4^ / well) and S17 cells (1.0 × 10^3^ / well), ALL-1 cells (1.0 × 10^4^ / well) alone, and S17 cells (1.0 × 10^3^ / well) alone were seeded on 96-well plates as indicated on day 1. Drug-library compounds were added on day 2. After 48 h, PDX cells were stained with Hoechst 33342 and PI. The viability of PDX cells was analyzed with an image analyzer. The growth of ALL-1 cells and S17 cells was measured by an MTT assay. **B.** Results of Cell-line screening. All compounds were plotted on a scattergram on which the relative MTT values of ALL-1 cells and S17 cells were set on the Y-axis and X-axis, respectively. The relative MTT values are relative values to the MTT value of the control cells treated with vehicle (DMSO). Effective compounds in PDX screening are plotted in red. Verteporfin is represented in green. The gray line indicates that the MTT value of ALL-1 is 0.5. Many of the red dots were plotted under the gray line, indicating that the effective compounds in PDX-cell screening were also frequently effective in Cell-line screening. **C.** Results of PDX-cell screening. All compounds were plotted on a scattergram on which the relative viabilities of PDX cells and relative MTT values of S17 cells were set on the Y-axis and X-axis, respectively. Effective compounds in Cell-line screening are plotted in red. Verteporfin is represented in green. The gray line indicates that the viability of PDX cells was 0.5. Few of the red dots were plotted under the gray line indicating that effective compounds in Cell-line screening were rarely effective in PDX-cell screening.

We performed drug screening using PhLO cells co-cultured with S17 cells as described previously [15] (PDX-cell screening), and compared the profiles of the selected drugs to those of screening using ALL-1 cells, a Ph^+^ ALL cell line (Cell-line screening). The library of 3440 compounds containing off-patent drugs was screened. Screening was performed using PhLO cells co-cultured with S17 cells, mono-cultured ALL-1 cells, and mono-cultured S17 cells, as shown in Figure 2A. All screenings were performed well with Z'-factors of 0.88, 0.56, and 0.93 and coefficient of variation values of 7.33%, 4.48%, and 2.26%, respectively.

All compounds were plotted on scattergrams, on which MTT values in ALL-1 cells (Figure 2B) or viabilities in PDX cells (Figure 2C) were set on the Y-axis and MTT values in S17 cells were set on the X-axis. Compounds with MTT values in ALL-1 cells (Figure 2B) or viabilities in PDX cells less than 0.5 were discriminated as effective compounds in each screening. Effective compounds in one screening were plotted with red dots in another screening (Figure 2B and 2C). The profiles of the selected drugs were markedly different between PDX-cell screening and Cell-line screening. Cell-line screening was generally more sensitive than PDX-cell screening. The number of effective compounds in PDX-cell screening was 60 (Figure 2C), whereas 597 compounds were effective in Cell-line screening (Figure 2B). Only 37 compounds out of 597 effective compounds (6%) in Cell-line screening were also effective in PDX-cell screening (Figure 2C), suggesting the difficulty associated with identifying effective compounds for PDX cells by Cell-line screening. On the other hand, 37 out of 60 effective compounds (62%) in PDX-cell screening were also effective in Cell-line screening.

The top 10 compounds in PDX-cell screening were shown in Table 1. We selected verteporfin for further analysis. Verteporfin exhibited strong cytotoxicity in PDX-cell screening, but did not show significant growth inhibition in Cell-line screening (Figure 2B and 2C). Verteporfin is an FDA-approved drug for the treatment of age-related macular degeneration (ARMD) and is suitable for being injected into humans.

**Table 1:** Top 10 drugs selected by PDX screening

	PhLO viability (%)	Relative MTT value of ALL1	Relative MTT value of S17
Pentoxifylline	0	0.46	0.87
Rilmenidine hemifumarate	0	0.66	0.75
AC-93253 iodide	0	0.29	0.83
Idarubicin	0	0.26	0.82
Chicago sky blue 6B	0	0.91	0.55
Pyrvinium pamoate	0	0.34	0.38
Aurantimycin A	0	0.21	0.29
Verteporfin Stattic	0.01 9.9	1.08 0.19	0.3 0.69

Furthermore, we made a new library by selecting the top 200 compounds in PDX-cell screening using PhLO cells and subjected them to other PDX-cell screenings using PhLH and PhLK cells and Cell-line screening using NPhA1, ALL-1, and TCC-Y/sr cells. All cells were PDX cells or cell lines of Ph^+^ ALL. PhLH and TCC-Y/sr both had the T315I mutation of BCR-ABL, which caused strong resistance to ABL kinase inhibitors. [7] The clinical and genetical backgrounds of the cell lines are summarized in Supplemental Table 3. We compared the drug sensitivity profiles of these cells. We evaluated drug effects in these Cell-line screenings with the same method as in PDX screenings, that is, with the image analyzer to make the assay conditions equal. The relationships between drug sensitivity profiles among PDX cells were weak, whereas those among cell lines were strong. The correlation coefficients between PhLO and PhLH, PhLO and PhLK, and PhLH and PhLK were 0.215, 0.375, and 0.554, respectively, while those between NPhA1 and ALL-1, NPhA1 and TCC-Y/sr, and ALL-1 and TCC-Y/sr were 0.817, 0.909, and 0.706, respectively (Supplemental Figure 1). These results suggested that PDX cells had more diverse drug sensitivity profiles than those of cell lines.

### Verteporfin had strong anti-leukemic effects through light-independent ROS production

**Figure 3:**
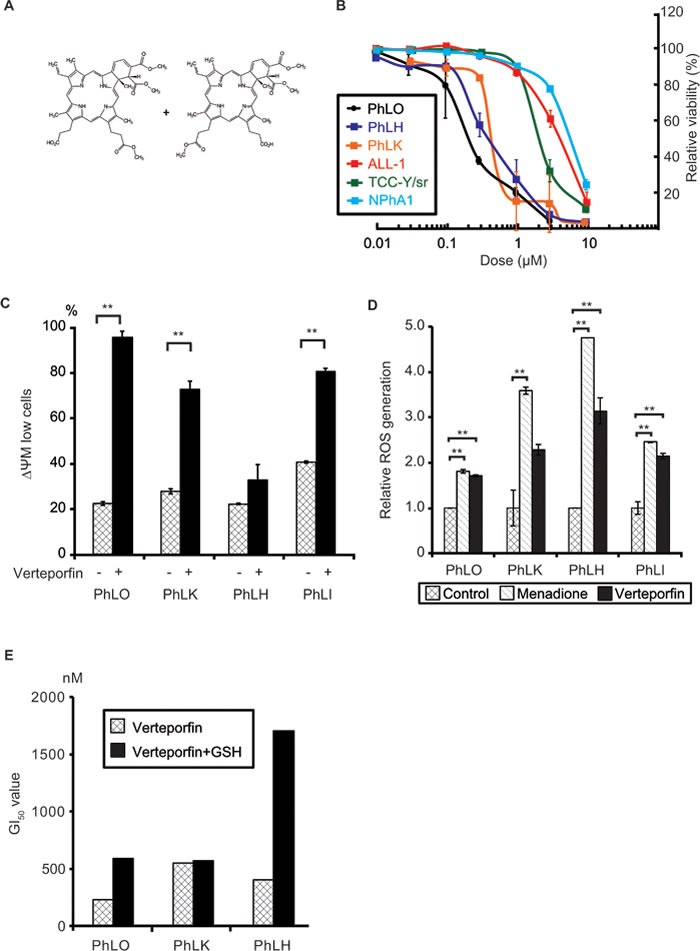
Verteporfin showed strong anti-leukemic effects through light-independent ROS production **A.** Chemical structure of verteporfin. **B.** Dose-dependent anti-leukemic effects of verteporfin on PDX cells and Ph^+^ ALL cell lines. PDX cells co-cultured with S17 cells and mono-cultured cell lines were treated with the indicated concentrations of verteporfin for 48 h. Viabilities were detected using a flow cytometer and presented as relative values to the viabilities of untreated cells. Each point represents the mean value for at least 3 independent experiments. Error bars show standard deviations. PDX cells were more sensitive to verteporfin than cell lines. **C.** Verteporfin reduced the mitochondrial membrane potential (ΔΨM) in PDX cells. The indicated PDX cells were treated with 1 μM verteporfin, labeled with JC-1 reagents, and analyzed with a flow cytometer. The ratio of low ΔΨM PDX cells was plotted on a bar graph. **D**. Verteporfin induced ROS production in PDX cells. The indicated PDX cells were incubated with verteporfin (2 μM) or menadione (50 μM) for 3 h. Menadione was used as the positive control of a ROS inducer. ROS production was measured by CellROX Green Oxidative Stress Reagents and plotted on a bar chart. **E.** GSH prevented the verteporfin-induced apoptosis in PDX cells. The indicated PDX cells co-cultured with S17 cells with or without 2 mM GSH in the culture medium were treated with the indicated dose of verteporfin as in **B.**. GI_50_ of verteporfin of the indicated PDX cells with or without GSH were determined and plotted on a bar chart. **: *p* < 0.001. GI_50_ were determined as results of at least 3 independent experiments. Error bars indicate standard deviations. GSH partly abolished the cytotoxicity of verteporfin in two of three PDX cells, suggesting that oxidative stress played a role in its cytotoxic effects.

The structural formula of verteporfin is shown in Figure 3A. Verteporfin is an example of a photosensitizer, which absorbs photons at specific wavelengths, induces the production of ROS, and then exerts cytotoxic effects. Verteporfin is currently used in photodynamic therapy for ARMD in which laser irradiation to the retina is performed 15 min after its intravenous administration in order to prevent angiogenesis. We compared the dose-dependency of its anti-leukemic effects among 3 PDX cells and 3 cell lines *ex vivo*. In addition to PhLO and ALL-1, PhLH, another Ph^+^ ALL PDX cell, and TCC-Y/sr, another Ph^+^ ALL cell line, were examined. PhLH and TCC-Y/sr both have the T315I mutation in BCR-ABL. PDX cells were more sensitive to verteporfin than cell lines. The concentrations to cause 50% growth inhibition (GI_50_) for PhLO, PhLH, and PhLK were 228 nM, 395 nM, and 538 nM, respectively, whereas GI_50_ for ALL-1, TCC-Y/sr, and NPhA1 were 3.93 μM, 2.11 μM, and 5.61 μM, respectively (Figure 3B). These results were consistent with the results of the screenings. Although the T315I mutation in BCR-ABL leads to strong resistance to ABL kinase inhibitors [7], it did not cause significant resistance to verteporfin.

Since we performed all these experiments under minimum white fluorescent light, the cytotoxicity observed was considered to be independent of light. In order to clarify the mechanisms underlying light-independent cytotoxicity, we examined the type of cell death induced by verteporfin, and found that it induced apoptosis in all 4 PDX cells (Figure 3C). We speculated that verteporfin produced ROS to some extent without light activation, which lead to apoptosis in PDX cells because of their high sensitivity to oxidative stress. We found that verteporfin produced ROS in a light-independent manner in all 4 PDX cells to the same extent as menadione, a well-known ROS producer among various cells [16] (Figure 3D). In order to further confirm the involvement of oxidative stress in verteporfin-induced cytotoxicity, we investigated the effects of glutathione (GSH), a major reducing agent in cells, on its cytotoxicity. GSH significantly reduced the sensitivity of 2 out of 3 PDX cells to verteporfin (Figure 3E), indicating the involvement of ROS production in the light-independent cytotoxicity of verteporfin.

### Verteporfin co-operatively worked with dasatinib *ex vivo* and *in vivo*

**Figure 4:**
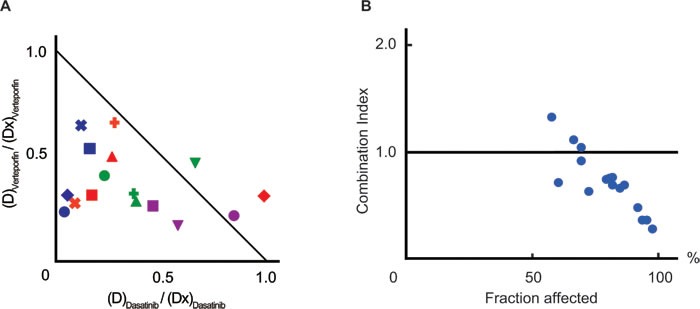
Verteporfin co-operated with dasatinib **A.** A normalized isobologram between verteporfin and dasatinib. PhLO cells co-cultured with S17 cells were treated with 16 combinations of verteporfin (60nM, 120nM, 180nM, and 240nM) and dasatinib (12nM, 24nM, 36nM, and 48nM). The viabilities of cells treated with each combination were measured using a flow cytometer after 48 h. Sixteen experimental data points are plotted. The list of markers of data points are shown in Supplemental Table 4. The normalized doses of dasatinib ((D)_Dasatinib_ / (Dx)_Dasatinib_) and verteporfin ((D)_Verteporfin_ / (Dx)_Verteporfin_) are shown on the Y-axis and X-axis, respectively. The diagonal line represents CI = 1.0, the line of additive effects. The plot below the diagonal line represents synergistic effect, and above represents antagonistic effects. **B.** Fraction affected-CI plot. CI values and dead cell ratios in **A.** were plotted on the Y-axis and X-axis of a scatter gram, respectively. CI values were less than 1.0 in most of the plots (CI mean, 0.73; CI range, 0.28-1.34), indicating synergistic anti-leukemic effects between verteporfin and dasatinib. Experiments performed in duplicate and mean values were plotted.

ABL kinase inhibitors are the main components of combined chemotherapies for Ph^+^ ALL, and dasatinib has been proposed as the most effective for Ph^+^ ALL. [17] Therefore, we determined whether verteporfin had synergistic effects with dasatinib. A normalized isobologram and fraction affected-combination index plot were made in order to estimate drug interactions. Most combination data points fell on the lower left area of the isobologram (Figure 4A) and most combination index values were less than 1.0 (mean, 0.73; range, 0.28-1.34, Figure 4B and Supplemental Table 4). These results indicated the existence of synergistic anti-leukemic effects between verteporfin and dasatinib.

**Figure 5:**
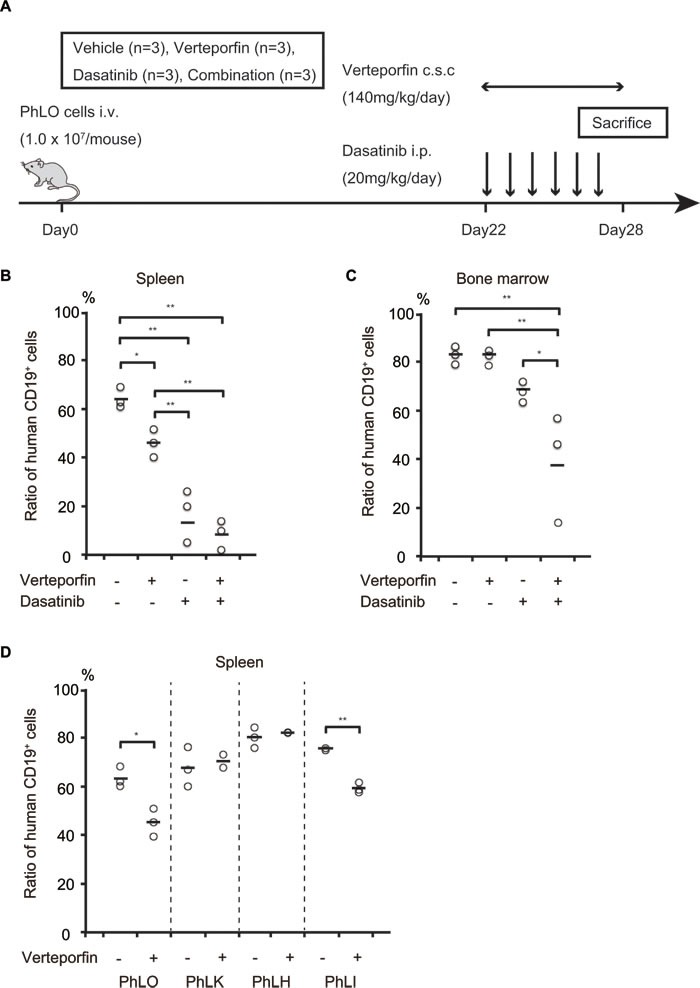
Verteporfin and dasatinib co-operated to reduce the number of leukemia cells *in vivo* **A.** Schematic presentation of the treatment schedule. NOG mice transplanted with PhLO cells were treated with vehicle, verteporfin, dasatinib, or both from days 22 to 28, as indicated. **B.** Leukemia cell number reductions in the spleen after the treatment with verteporfin and dasatinib. The leukemia cell ratio in spleen cells was analyzed using a flow cytometer with anti-human CD19 antibodies. Leukemia cell ratios in all mice were plotted. Single therapies with verteporfin and dasatinib significantly reduced the leukemia cell ratio. **C.** Leukemia cell number reductions in the bone marrow after the treatment with verteporfin and dasatinib. The leukemia cell ratio in bone marrow cells was analyzed as in **B.**. The combination therapy showed significantly enhanced effects, indicating synergistic effects between verteporfin and dasatinib *in vivo*. **D.** Comparison of *in vivo* effect of verteporfin among 4 PDX models. NOG mice were transplanted with the indicated PDX cells were treated with vehicle or verteporfin as in **A.**. Leukemia cell ratio in spleen was analyzed as in **B.**. **: *p* < 0.001, *: *p* < 0.05. The horizontal line is the mean of measurements. Abbreviations, i.v.: intravenous injection; c.s.c.: continuous subcutaneous injection; i.p.: intraperitoneal injection.

We next examined the anti-leukemic effect of verteporfin *in vivo*. In clinical use for ARMD, verteporfin is administered at a maximum of once a week. Therefore, in the first trial, we administered verteporfin (10 mg/kg) on alternate days from days 14 to 28 after the transplantation of leukemia cells; however, it did not lead to significant reductions in the number of leukemia cells (data not shown). Since its blood concentrations were very low, 3.7 nM to 21.8 nM, 24 h after its administration at 12.5 mg/kg (Supplemental Figure 2A), continuous infusion appeared to be required in order for it to exert its anti-leukemia effects. We used osmotic pumps that delivered drugs by an osmotic process at a controlled rate for continuous infusion. The mean blood concentration of verteporfin after the implantation of osmotic pumps was 654 nM, approximately 3 folds higher than its GI_50_ in the *ex vivo* experiments (Supplemental Figure 2B). We assessed the *in vivo* effects of verteporfin using this system. Twelve NOG mice transplanted with PhLO cells were treated with vehicle, verteporfin, dasatinib, or a combination of both from days 22 to 28, as shown in Figure 5A. The body weights of mice were similar among each group on day 28, suggesting that drug toxicity was not severe in any group (Supplemental Figure 2C). Single therapies with verteporfin and dasatinib significantly reduced the leukemia cell ratio, and combined therapy further reduced the number of leukemia cells in the spleen (Figure 5B). Both of the single therapies had weaker anti-leukemic effects in bone marrow than in the spleen, however the combination therapy showed significantly enhanced effects (Figure 5C). These results indicated that verteporfin exhibited anti-leukemic activity in Ph^+^ ALL when administered by itself and also in combination with dasatinib *in vivo*. We further investigated the effect of verteporfin on other 3 PDX models. Significant reduction of leukemia cells in spleen was observed in PhLI mice, whereas the effect was not significant in PhLK and PhLH mice (Figure 5D), suggesting sensitivity of verteporfin were various *in vivo*. We finally examined the effect of verteporfin on normal hematopoiesis and found that verteporfin did not significantly reduce the number of peripheral blood cells and bone marrow mononuclear cells (Supplemental Figure 3A and 3B), supporting the safety of verteporfin.

## DISCUSSION

Using PDX-cell screening, we herein demonstrated that verteporfin exerted strong anti-tumor effects on Ph^+^ ALL. It produced ROS in leukemia cells and induced apoptosis, which was a different mechanism of action from conventional anti-tumor drugs. This drug had weak anti-leukemic effects on cell lines in spite of its strong anti-leukemic effects on PDX cells. Thus, Cell-line screening did not select this drug as a candidate anti-leukemic drug. This is an exceptional case. Cell-line screening was more sensitive than PDX-cell screening and discriminated many (597) compounds as effective; however, 94% of these compounds were not effective for PDX cells (Figure 2C). This was markedly different from 62% of the effective compounds in PDX-cell screening also being effective in Cell-line screening (Figure 2B). Even when effective compounds were strictly selected, that is, only compounds that inhibited growth by more than 80% being selected as effective compounds in Cell-line screening, only 11 (12%) out of 93 effective compounds were also effective in PDX-cell screening (data not shown). In addition, 23 compounds including verteporfin were effective in PDX cell screening, but were not in Cell-line screening. Although some of these differences in drug sensitivity may have been due to differences between the primary leukemia cells of ALL-1 cells and PhLO, based on the molecular pathological homogeneity of Ph^+^ ALL, many of these differences may be attributed to differences between PDX cells and cell lines. These results showed the difficulty in selecting the effective compounds for primary tumor cells by Cell-line screening and the superiority of PDX-cell screening in the development of anti-tumor drugs. Of note, the result that PDX cells had more diverse drug sensitivity profiles than those of cell lines (Supplemental Figure 1) was important. Since cell lines are tumor cells that are strongly selected based on growth rates under *ex vivo* culture, cell lines may lose the diversity of drug sensitivity during the establishment of cell lines from primary tumor cells. These results indicated the difficulty of choosing effective compounds for primary tumor cells by Cell-line screening and the superiority of PDX-cell screening in the development of anti-tumor drugs. The best method to search for compounds that commonly show strong anti-tumor effects in multiple PDX cells may be by multiple PDX-cell screenings.

We cannot currently conclude that PDX-cell screening is superior, because we need to determine whether this screening has the ability to select drugs more efficiently that have strong anti-tumor effects on actual cancer cells in patients. PDX-cell screening selects anti-cancer reagents that cannot be identified by Cell-line screening, and provides new candidates for anti-cancer drug development. PDX-cell screening may detect anti-cancer drugs among existing drugs that have been overlooked for their anti-tumor activities.

We revealed that the light-independent anti-leukemic effects of verteporfin were attributed to ROS production without light. Pyruvinium pamoate, a ROS inducer, was also selected by PDX-cell screening using lymphoma PDX cells and exhibited strong anti-lymphoma activity in PDX mice in our previous study. Pyruvinium pamoate, similar to verteporfin, had strong anti-leukemic effects on PDX cells, but only had moderate effects on ALL-1 in this screening (Table 1). These results indicated that PDX cells were more sensitive to oxidative stress than cell lines. Reducing agents such as 2-mercaptoethanol are often added to culture media during the early stage of the establishment of cell lines including ALL-1. [18] The addition of reducing agents becomes unnecessary after the establishment of cell lines in many cases, suggesting that cell lines obtain resistance to oxidative stress during their establishment. PDX-cell screening may be a useful tool for discovering oxidative-stress-inducing drugs that are hard to select using Cell-line screening.

Verteporfin is activated by light, most efficiently at a wavelength of 690 nm, and produces ROS in cells. Its cytotoxicity for some cancer cells such as pancreatic cancer, breast cancer, and leukemia has been reported; however, its cytotoxicity had been activated by light in all these cases. [19–21] Therefore, pioneering cancer therapy using verteporfin has been localized therapy combined with photoirradiation. [19] This is the first study on the light-independent cytotoxicity of verteporfin. This effect has been overlooked for a long period of time despite intensive investigations being conducted on this drug, possibly because it had weak cytotoxicity for cell lines and its cytotoxicity for primary tumor cells was hard to estimate. This result will promote the application of this drug to systemic chemotherapy for cancer and may markedly expand its potential uses.

Verteporfin was originally designed to be excreted rapidly after photoirradiation in order to prevent side effects such as photodermatosis. Therefore, the half-life of this drug is short at 5.6 h. Verteporfin is an FDA-approved drug for ARDM; however, its approved usage is for single intravenous administration. Repetitive administration is only permitted one time with an interval at least one week. Since verteporfin did not exert its anti-leukemic effects *in vivo* with an alternate day administration protocol (data not shown), non-approved usage such as continuous infusion is needed when verteporfin is applied to the treatment of leukemia. No safety information for such usage is currently available, and therefore, a phase I study is needed.

The anti-leukemic effects of this drug *in vivo* were not as strong as those of dasatinib, and this drug was not effective in 2 out of 4 PDX models, PhLK and PhLH (Figure 5D). These will be because the blood concentration of verteporfin was not very high, only approximately 3-fold higher than its GI_50_ for PhLO cells. GI_50_ for PhLK and PhLH were about 2.4 and 1.7 times higher than PhLO (Figure 3B), which would lead to verteporfin-resistance of these mice model. Due to the poor solubility of verteporfin and the limited volume of the osmotic pump (200 μl), we were unable to administer more in this system. The development of a better method for dissolving verteporfin will enable higher blood concentrations and stronger anti-leukemic effects *in vivo*.

Verteporfin had synergistic anti-leukemic effects with dasatinib. Previous studies reported the involvement of ABL kinase in resistance to oxidative stress. The nuclear translocation of c-ABL and activation of early growth response protein 1 by c-ABL have been shown to contribute resistance to oxidative stress. [22, 23] A previous study demonstrated that adaphostin-induced oxidative stress overcame resistance to imatinib, another clinically used ABL kinase inhibitor, in Ph^+^ ALL. [24] The inhibition of ABL kinase by dasatinib may attenuate the tolerance of leukemia cells to oxidative stress, which may function co-operatively with verteporfin-induced oxidative stress. The uptake of verteporfin by the primary cells of CML, another leukemia developed by BCR-ABL, is greater than that by the normal mononuclear cells of peripheral blood and bone marrow, which further explains their co-operation *in vivo*.

In summary, we herein performed PDX-cell screening using the PDX cells of Ph^+^ ALL, identified oxidative stress as an important anti-leukemic mechanism that co-operatively functions with ABL kinase inhibition, and discovered verteporfin as an anti-leukemic ROS inducer. By introducing the phenotype of primary cancer cells at the earlier *in vitro* stages of drug development, PDX-cell screening sheds new light on anti-cancer drug development.

## MATERIALS AND METHODS

### Cells

S17 cells, a murine bone marrow stromal cell lines, were previously described. [14] S17 cells were cultured in Roswell Park Memorial Institute medium (RPMI) supplemented with 10% fetal bovine serum (FBS) for maintenance. ALL-1 cells, TCC-Y/sr cells, and NPhA1 were maintained in 10% FBS-containing Iscove's modified Dulbecco's medium (ISCOVE) and 10% FBS-containing RPMI medium, respectively. [25, 26] All cell lines have not been tested for authentication in our laboratory.

### Reagents and antibodies

Verteporfin and reduced-form GSH were purchased from Wako Pure Chemical Industries (Osaka, Japan). Dasatinib and menadione was obtained from Chemscene (Princeton, NJ, USA) and Sigma Aldrich (St. Louis, MO, USA), respectively. An anti-human CD19 antibody and anti-mouse CD45 antibody were purchased from BD Biosciences (San Jose, CA, USA).

### Compound library

A library of 3440 compounds mainly consisting of off-patent drugs and pharmacologically active reagents was kindly provided by The Drug Discovery Initiative (The University of Tokyo, Tokyo, Japan) and described previously. [15]

### Establishment of PDX cells

PhLO and PhLK cells were established in a previous study. [12] PhLH and PhLI were established as described previously. [12, 14, 15] Briefly, primary Ph^+^ ALL cells (6.8-20 × 10^6^ cells) were injected into the tail vein of non-irradiated 8-week-old male NOG mice. To prevent human T cell expansion, 0.1 mg anti-CD3 antibody (Janssen Pharmaceutical, Tokyo, Japan) was injected on the same day. For the *in vivo* passage of PDX cells, BM cells or spleen cells (5 × 10^6^ cells) from a PDX mouse were injected into another NOG mouse with an 8-week interval. Primary cells were collected from patients after obtaining written informed consent, and the Institutional Review Board of Nagoya University Graduate School of Medicine approved this study.

### PDX-cell screening

S17 cells (1.0 × 10^3^ /well) and PhLO cells (1.0 × 10^4^ /well) were seeded on 96-well plates in 10% FBS-containing RPMI medium on day 1. On day 2, the library compounds (2 μM each) were added to each well. After 48 hours (h) on day 4, total and dead PDX cells were stained with Hoechst 33342 and Propidium Iodide (PI), respectively. Total and dead PDX cells were counted separately from S17 cells using an Array Scan VTI HCS image analyzer (Thermo Fisher Scientific, Waltham, MA, USA) as described previously. [15] Since the library contained some photosensitive compounds, all experiments were performed under conditions that avoided sunlight and laser light.

### Cell-line screening

ALL-1 cells (1.0 × 10^4^ /well) and S17 cells (1.0 × 10^3^ /well) were seeded on 96-well plates on day 1. Library compounds were added to each well on day 2. MTT assays were performed using Cell Counting Kit-8 reagent (Dojindo Laboratories, Kumamoto, Japan) on day 4.

### Detection of apoptosis and ROS generation

These assays were performed as described previously. [15]

### Estimation of drug interactions

PDX cells co-cultured with S17 cells were treated with 16 combinations of verteporfin (60 nM, 120 nM, 180 nM, and 240 nM) and dasatinib (12 nM, 24 nM, 36 nM, and 48 nM). The viabilities of cells treated with each combination were measured after 48 h using FACS Aria flow cytometer (BD Biosciences, San Jose, CA, USA). In order to estimate drug interaction between verteporfin and dasatinib, a normalized isobologram and fraction affected-combination index (CI) plot were made using CompuSyn software (ComboSyn, Paramus, NJ, USA). [27] CI values greater than 1.0 indicated antagonistic effects, equal to 1.0 additive effects, and below 1.0 synergistic effects.

### Estimation of *in vivo* drug effects

PhLO cells (1.0 × 10^7^ /mouse) were injected intravenously into 6-week-old male NOG mice, which were then treated with vehicle, verteporfin (140 milligram (mg)/kilogram (kg)/day), dasatinib (20 mg/kg/day), and a combination of these drugs from days 22 to 28. Verteporfin was administered by continuous subcutaneous infusion (c.s.c.) using Alzet osmotic pumps (Alzet, Cupertino, CA, USA). An intraperitoneal injection (i.p.) was performed for dasatinib. All mice were sacrificed on day 28 and the chimerism of leukemia cells was investigated by flow cytometer using an anti-human CD19 antibody and anti-mouse CD45 antibody. Blood concentrations of verteporfin were calculated by LCMS-2020 (Shimadzu Corporation, Kyoto, Japan). All animal experiments were approved by the Nagoya University Animal Ethics Committee.

### Statistical analyses

Differences between two groups were analyzed with the Student's *t*-test. Differences between more than three groups were examined with the Tukey-Kramer test. Statistical analyses were performed by R software (R Foundation for Statistical Computing, Vienna, Austria) and *p* < 0.05 was considered significant.

## SUPPLEMENTARY MATERIALS FIGURES AND TABLES


