# Supplementary material for: The photosensitizer verteporfin has light-independent anti-leukemic activity for Ph-positive acute lymphoblastic leukemia and synergistically works with dasatinib
Source: Oncotarget. 2016 Aug 2;7(35):56241–52. doi: 10.18632/oncotarget.11025 (PMC5302911; doi:10.18632/oncotarget.11025)
Supplement: Supplementary file 1 [file oncotarget-07-56241-s001.pdf]

The photosensitizer verteporfin has light-independent anti-leukemic activity for Ph-positive acute lymphoblastic leukemia and synergistically works with dasatinib

Supplementary Material

Supplemental Table 1. Characteristics of Ph^+ ALL patients.

Name	PhLO	PhLK	PhLH	PhLI
Disease	Ph^+ALL	Ph^+ALL	Ph^+ALL	Ph^+ALL
Disease status	Initial stage	Relapse	Relapse	Initial stage
Age	68	77	60	29
Gender	F	F	M	M
ABL mutation	Wild	Y253H	T315I	Wild
Karyotype abnormalities in patients	46, XY, t(9;22)(q34;q11.2)	46, XX, t(2;7)(p11.2;p13), t(9;22)(q34;q11.2)	46, XY, t(9;22)(q34;q11.2), t(14;20)(q32;q13.1)	46, XY, t(9;22)(q34;q11.2)

Abbreviations, Ph^+ ALL: Philadelphia chromosome positive acute lymphoblastic leukemia; F: female; M: male.

Supplemental Table 2

Characteristics of Ph+ ALL PDX.

Name	PhLO	PhLK	PhLH	PhLI
Number of injected cells	1.0×10^7	1.2×10^7	6.8×10^6	2.0×10^7
Number of obtained cells (8 weeks, per mouse)	5.8×10^8	1.3×10^8	2.1×10^8	1.7×10^8
Leukemia chimerism in NOG mouse spleen (%)	95.7	86.0	92.5	92.7

Supplemental Table 3. Characteristics of Ph^+ ALL cell lines

Name	ALL1	TCC-Y/sr	NPhA1
Disease	Ph^+ALL	Ph^+ALL	Ph^+ALL
Disease status	Initial stage	ND	Initial stage
Age	6	ND	68
Gender	F	ND	F
ABL mutation	ND	T315I	Wild
Karyotype abnormalities in patients	46, XY, t(9;22)(q34;q11.2)	46, XX, t(9;22)(q34;q11.2)	46, XY, t(9;22)(q34;q11.2),

Abbreviations, Ph^+ ALL: Philadelphia chromosome positive acute lymphoblastic leukemia; F: female; M: male; ND: not determined.

Supplemental Table 4. List of markers of data points in normalized isobologram and CI plot.

	Verteporfin (µM)	Verteporfin (normalized)	Dasatinib (µM)	Dasatinib (normalized)	Fa	CI
●	0.24	0.24	0.048	0.04	0.97	0.28
■	0.18	0.32	0.048	0.17	0.91	0.49
▲	0.12	0.29	0.048	0.37	0.84	0.66
▼	0.06	0.17	0.048	0.58	0.79	0.75
◆	0.24	0.31	0.036	0.06	0.95	0.37
✖	0.18	0.28	0.036	0.09	0.93	0.37
✚	0.12	0.32	0.036	0.37	0.81	0.69
●	0.06	0.21	0.036	0.84	0.69	1.05
■	0.24	0.53	0.024	0.16	0.86	0.69
▲	0.18	0.50	0.024	0.27	0.80	0.77
▼	0.12	0.46	0.024	0.67	0.66	1.13
◆	0.06	0.29	0.024	1.04	0.57	1.33
✖	0.24	0.63	0.012	0.12	0.81	0.75
✚	0.18	0.64	0.012	0.28	0.69	0.92
●	0.12	0.40	0.012	0.23	0.72	0.63
■	0.06	0.26	0.012	0.46	0.60	0.72

Abbreviations, Fa: Fraction affected; CI: Combination index

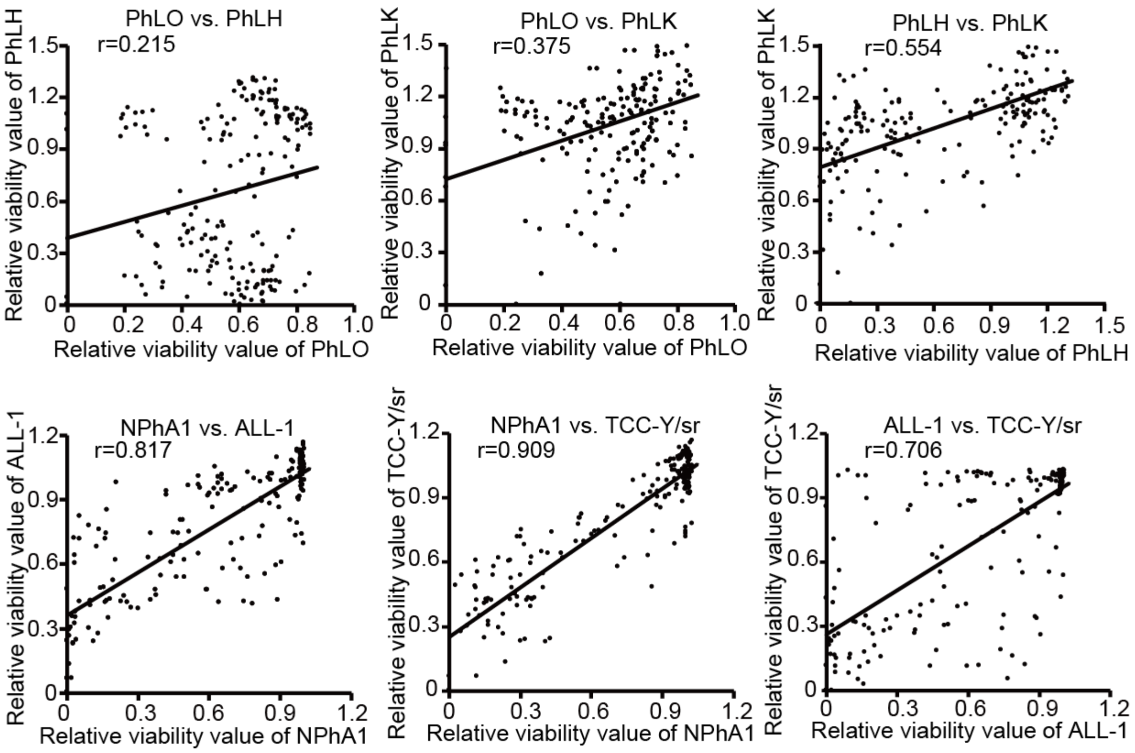

Supplemental Figure 1. PDX cells had more diverse drug sensitivity profiles than those of cell lines. The top 200 compounds selected by PDX screening using PhLO cells were subjected to PDX-cell screening using PhLH and PhLK cells and Cell-line screening using NPhA1, ALL-1, and TCC-Y/sr cells. Drug effects in Cell-line screenings were analyzed with the image analyzer as in PDX screenings. The compounds are plotted on a scattergram in which the relative viabilities of the indicated PDX cells (upper 3 panels) or relative MTT values of the indicated cell lines (lower 3 panels) are set on the Y-axis and X-axis, respectively. The linear trendline and correlation coefficient (*r*) are shown. The relationships between drug sensitivity profiles among PDX cells were weak, whereas those among cell lines were strong.

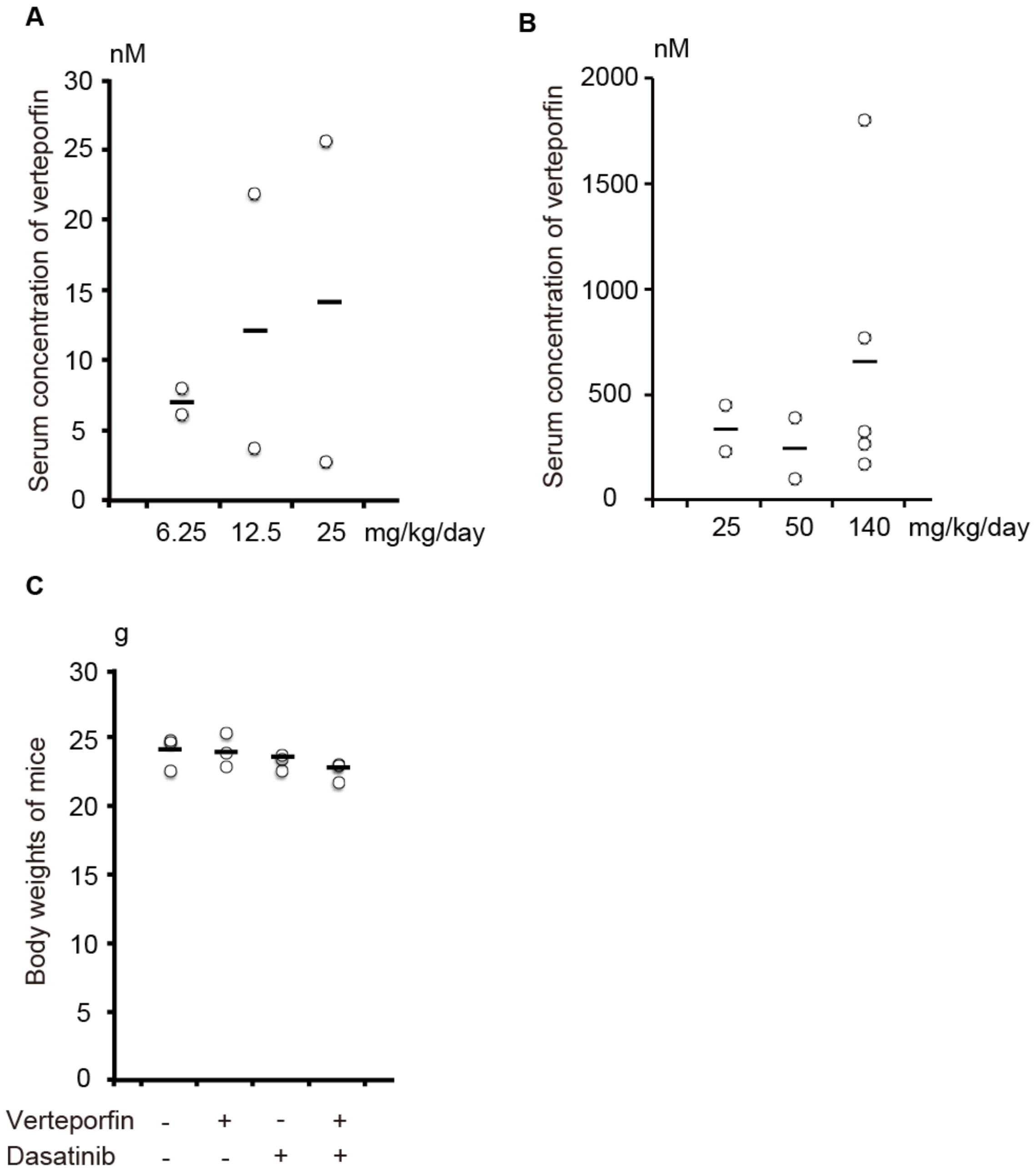

Supplemental Figure 2. (A) Single administration of verteporfin could not keep its blood concentration at effective level for 24h. Mice (two per each group) were administered indicated dose of verteporfin and sacrificed 24h later for blood collection. Serum drug concentrations are plotted. Horizontal lines represent the mean values. (B) Blood concentration of verteporfin during continuous administration. Mice (two per each group) were continuously administered verteporfin at indicated dose using osmotic pumps for 7 days administration. Mice were sacrificed for blood collection on day 3 when the blood concentration

was expected to reach to its plateau phase. Serum drug concentrations are plotted. Horizontal lines represent the mean values. (C) Continuous administration of verteporfin did not cause significant body weight loss. Body weights of all mice in Figure 5 on sacrificed day are shown. Horizontal lines represent the mean values.

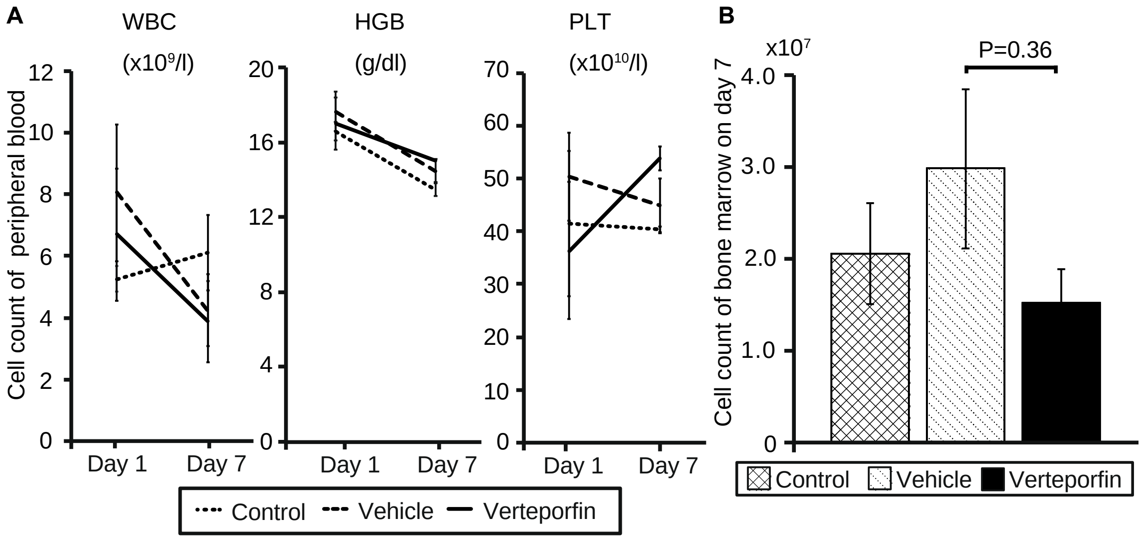

Supplemental Figure 3. Effects of verteporfin on normal hematopoiesis. (A) Changes in blood cell counts after the verteporfin treatment. Ten-week-old Balb/c mice were untreated (Control: n=2) or were implanted with osmotic pumps filled with DMSO (Vehicle: n=2) or verteporfin (Verteporfin: n=2). Blood cell counts were performed on Day 1 (after the implantation of pumps) and Day 7. Higher WBC counts in Vehicle mice on Day 1 were attributed to a reaction to the stress caused by the implantation of pumps. Decreases in RBC in all mice were expected to be due to the effects of blood collection on Day 1. Changes in blood cell counts in Verteporfin mice were similar to those in Control and Vehicle mice. (B) Changes in bone marrow mononuclear cell (MNC) counts after the verteporfin treatment. MNC in the bone marrow of mice in (A) were counted on Day 7 and plotted on bar charts as relative values to Control mice. MNC counts in Verteporfin mice were not significantly lower than those in Vehicle mice.
